# Prediction Potential of Serum miR-155 and miR-24 for Relapsing Early Breast Cancer

**DOI:** 10.3390/ijms18102116

**Published:** 2017-10-10

**Authors:** Petra Bašová, Michal Pešta, Marek Sochor, Tomáš Stopka

**Affiliations:** 1 BIOCEV, First Faculty of Medicine, Charles University, Vestec 25250, Czech Republic; basova.petra@gmail.com; 2 Faculty of Mathematics and Physics, Charles University, Prague 18675, Czech Republic; pesta@karlin.mff.cuni.cz; 3 Comprehensive Cancer Centre, Regional Hospital Liberec, Liberec 46063, Czech Republic; sochor.marek73@gmail.com

**Keywords:** breast cancer, microRNA, relapse, miR-155, miR-24, Ki-67

## Abstract

Oncogenic microRNAs (oncomiRs) accumulate in serum due to their increased stability and thus serve as biomarkers in breast cancer (BC) pathogenesis. Four oncogenic microRNAs (miR-155, miR-19a, miR-181b, and miR-24) and one tumor suppressor microRNA (let-7a) were shown to differentiate between high- and low-risk early breast cancer (EBC) and reflect the surgical tumor removal and adjuvant therapy. Here we applied the longitudinal multivariate data analyses to stochastically model the serum levels of each of the oncomiRs using the RT-PCR measurements in the EBC patients (*N* = 133) that were followed up 4 years after diagnosis. This study identifies that two of the studied oncomiRs, miR-155 and miR-24, are highly predictive of EBC relapse. Furthermore, combining the oncomiR level with Ki-67 expression further specifies the relapse probability. Our data move further the notion that oncomiRs in serum enable not only monitoring of EBC but also are a very useful tool for predicting relapse independently of any other currently analyzed characteristics in EBC patients. Our approach can be translated into medical practice to estimate individual relapse risk of EBC patients.

## 1. Introduction

Early breast cancer (EBC) represents an early stage tumorous process defined as a breast cancer (BC) (including ductal carcinoma in situ and invasive BC stages I, IIA, IIB and IIIA) that has not spread beyond the breast or the axillary lymph nodes however, that can progress into a deadly disseminated cancer. There exist standard risk factors, including expression of hormonal receptors, HER2, Ki-67, and grade, none of which are fully reliable for predicting relapse. Therefore, identification of reliable diagnostic and prognostic biomarkers is one of the most important tasks in current EBC research. MicroRNAs appear as very promising tumor biomarkers [1], mostly because they are highly resistant against degradation in many different tissues and body fluids, are easy to determine, and most importantly, their level is often a measure of the tumor burden associated with other clinically relevant characteristics of the oncogenic process. Circulating microRNAs are 18–24 nt long non-coding RNAs that are present in the serum or plasma as a result of higher turnover from the malignant cells [2]. Within these cells microRNAs bind mRNA molecules and inhibit gene expression by posttranscriptional mechanisms involving degradation of mRNA and inhibition of translation.

Previously, several oncomiRs were shown to display increased abundance and to mediate pathogenesis of BC including increased levels of miR-155 [3], miR-19a [4] miR-181b [5], or miR-24 [6] in the tumor tissue. There also exist microRNAs with tumor suppressive roles in BC including let-7a [7] that are suitable denominator controls for qPCR analysis of serum microRNA [8]. The relatively stably low levels of let-7a were reported by additional study that utilized miR-16 as a control for qPCR [9]. We have followed up on this and previously devised a monitoring technique utilizing detection of these five miRs in three consecutive serum samples, before and throughout the treatment, of 63 EBC patients. Our data showed that the expression of four BC pathogenesis-related oncomiRs (miR-155, miR-19a, miR-181b, and miR-24) was increased at diagnosis of EBC patients and declined following the combined therapy both in primary tumor tissues as well as in the sera [10]. Upregulation of miR-24 was independently observed in the BC sera elsewhere [6]. Interestingly, levels of miR-155 were also elevated in the BC patient sera especially in those with metastatic spread [11]. While high-risk EBC patients showed notably delayed and less-pronounced decrease of oncomiR expression following the surgical tumor removal, the relapse was characterized by their re-expression [10]. Thus, levels of microRNAs can be potentially very useful for identifying patients with increased relapse risk.

Based on the above-mentioned points we have closely followed the EBC patients for more than 4 years and using standard criteria assessed their clinical state as well as recorded the occurrence of a relapse. In addition, we have doubled the number of EBC patients (and oncomiRs detection measurements) and applied the multivariate data analysis model to evaluate the roles of oncogenic microRNAs in predicting EBC relapse. Our data provide the following results: two of the studied oncomiRs at early clinical time points possess predictive power foretelling the relapse of BC as well as represent an independent risk factor that can be combined with a standard risk-factor to improve the relapse-prediction accuracy.

## 2. Results

### 2.1. OncomiRs miR-155 and miR-24 Are Predictive of Early Breast Cancer (EBC) Relapse

**Figure 1:**
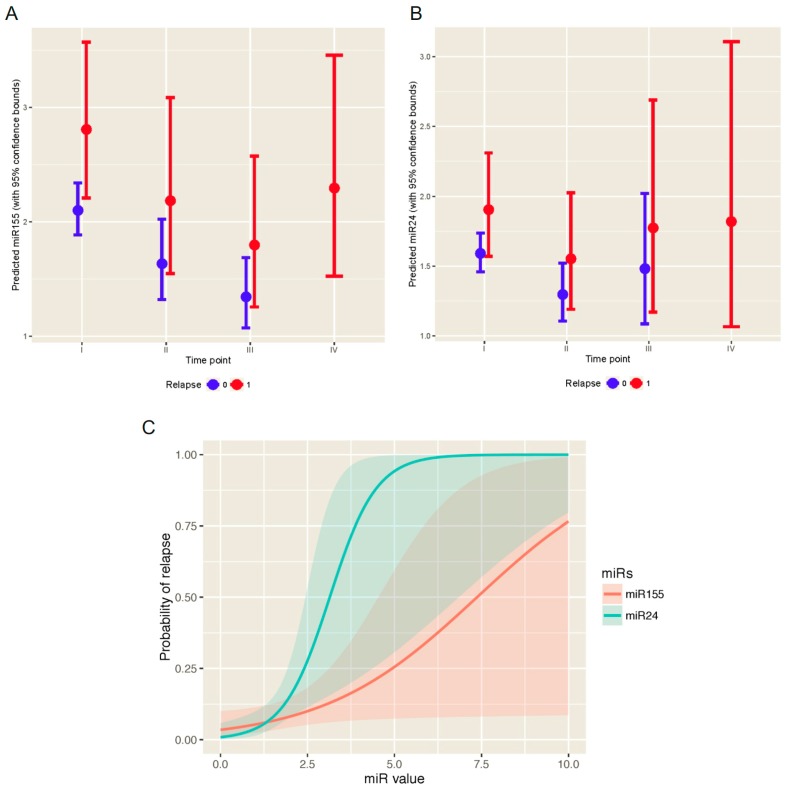
MiR-155 and miR-24 were predictive of the EBC relapse (*p*-values 0.025 and 0.041, respectively). Predicted oncomiR values of miR-155 (**A**) and miR-24 (**B**) over the time points (I–IV) with 95% prediction bands for the relapsed (red intervals, 1) and non-relapsed (blue intervals, 0) patients based on the GEE model; (**C**) MiR-155 and miR-24 increase the probability of relapse (*p*-values 0.028 and 0.020, respectively); 95% confidence bands of relapse are also shown.

We determined the levels of oncomiRs (miR-155, miR-19a, miR-181b, and miR-24) in sera of 133 patients relative to let-7a (see M&M section) and obtained a set of values from each time point: one day prior to a surgical tumor removal operation (time point I), 14-28 days after surgical operation (time point II), and 14-28 days after first non-surgical treatment modality: either chemotherapy or radiotherapy (time point III) as well as in case of relapse (time point IV). These values (as shown in Figure S1) suggested that relapsing patients expressed at the time points II-IV markedly increased oncomiR (miR-155, miR-24) levels. In order to statistically evaluate the data, we constructed and used longitudinal multivariate data analysis, specifically the generalized estimating equations (GEE) model (see Statistical analysis within the M&M section), for each of the oncomiRs. This enabled the analysis of a particular oncomiR level within EBC patient serum with respect to a time point and EBC relapse. Additionally, we aimed to define the expected level of each oncomiR at each time point. The multivariate GEE model revealed that miR-155 and miR-24 were predictive of the EBC relapse (*p*-values 0.025 and 0.041, respectively). The predicted oncomiR values over the time points for the relapsed and not relapsed patients are shown in Figure 1A,B. MiR-155 levels of the relapsed patients in sera were elevated already at diagnosis (time point I) and gradually decreased at time points II and III; between time points II and III the predicted value of miR-155 was 1.22 times lower. At relapse (time point IV) the level of miR-155 increased and exceeded (1.05 fold) the time point II. Mir-24 levels in the relapsed patients decreased markedly at the time point II from the elevated level at diagnosis. At time points III and IV the predicted miR-24 values increased 1.14 and 1.17 times respectively. Moreover, the predicted miR-24 serum levels were 1.34 times higher for the relapsed patients compared to the non-relapsed ones (significant *p*-value 0.025) in each relevant time point (i.e., I, II, and III). For the miR-24, the predicted oncomiR values were 1.20 times higher for the relapsed patients compared to the non-relapsed ones (borderline *p*-value 0.086). Furthermore, there is a significant impact of the time point II measurement of miR-155 and miR-24 on the probability of relapse (*p*-values 0.028 and 0.020, respectively). The predicted probability of relapse with respect to miR-155 and miR-24 at time point II can be obtained using the generalized linear model (GLM) with Bernoulli distributed response and are shown in Figure 1C. On the other hand, miR-19a and miR-181b (Figure S2) are statistically insignificant with respect to the EBC relapse (*p*-values 0.250 and 0.240, respectively). In addition, there is no effect of miR-19a and miR-181b measured after the operation (time point II) on the probability of relapse (*p*-values 0.054 and 0.062, respectively). Taken together, the values of miR-155 and miR-24 are elevated in relapsed patients compared to non-relapsing patients in each time point. The probability of relapse can be obtained utilizing the GLM model (Figure 1C).

### 2.2. Ki-67 Expression Specified the Relapse Probability Defined by Levels of miR-155 or miR-24

**Figure 2:**
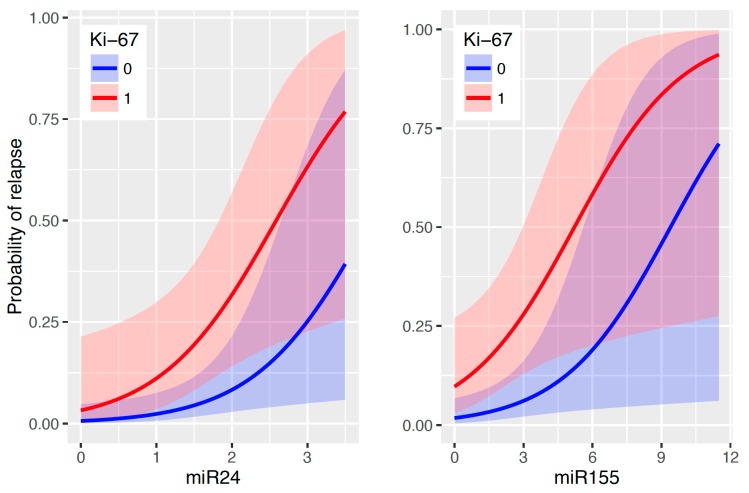
Predicted probability of relapse with respect to the values of miR-155 and miR-24 at time point II and according to Ki-67 positivity (>20% equals to Yes = 1, No = 0 equals to <20%) displayed together with 95% confidence bands. *p*-value = 0.013 in case of miR-155/Ki-67 and *p*-value = 0.023 in case of miR-24/Ki-67.

Besides the effects of the oncomiRs levels determined immediately after the surgical removal of the tumor, risk characteristics like triple-negativity, HER2, grade II, and axillary lymphadenopathy as well as type of therapy or family history have no effect on the probability of relapse. A possible explanation is that the time point II measurement of miR-155 and/or miR-24 provides a more precise and/or a more informative view with respect to BC relapse compared to the above-mentioned risk characteristics. Their effect is hence not significant (all the *p*-values above 0.05) when the oncomiRs are simultaneously taken into an account. The only clinical risk factor that influences the relapse chance (alongside miR-155 and miR-24) is Ki-67 (*p*-value 0.013 in case of miR-155 and *p*-value 0.023 in case of miR-24). Therefore, Ki-67 (positivity of more than 20% of cells) makes the prediction of relapse more precise when also taking into an account the measurements of the oncomiRs (Figure 2). The GLM approach provides prediction formulas to determine the probability of relapse, which can be split in the following three cases:

*a*. Only miR-155 level is available (or considered)
  $$Probability[Relapse_{i}]=1/[1+\exp\{4.019-0.428(\mathrm{miR-}155{\mathrm{II}}_{i})-1.788(\mathrm{Ki-}67_{i}\geq 20\%)\}]$$
*b*. Only miR-24 level is available
  $$Probability[Relapse_{i}]=1/[1+\exp\{5.023-1.311(\mathrm{miR-}24{\mathrm{II}}_{i})-1.633(\mathrm{Ki-}67_{i}\geq 20\%)\}]$$
*c*. Both miR-155 and miR-24 levels are available
  $$Probability[Relapse_{i}]=\frac{1}{\left[1+\exp\{5.687-0.396(\mathrm{miR-}155{\mathrm{II}}_{i})-1.206(\mathrm{miR-}24{\mathrm{II}}_{i})-1.605(\mathrm{Ki-}67_{i}\geq 20\%)\}\right]}$$

Here for instance, miR-155II*_i_* means the miR-155 measurement at time point II for the *i*th patient. As a practical application and usage of these prediction formulas, let us consider a patient with Ki-67 < 20% and only available miR-24 measurement after operation (measured value 2.00), then the predicted probability of relapse is 8.31% (according to *b*). Furthermore, for a patient with Ki-67 ≥ 20% we aim to know the value of miR-155 at time point II such that the probability of relapse is at least 90%. According to *a*, we end up with 10.3. Taken together, using standardly determined status of Ki-67 accompanied by levels of two oncomiRs (miR-155, miR-24) would allow us to define probability of relapse as well as to define level of the oncomiRs in respect to relapse probability. The estimated quantitative effects of the risk characteristics from the previously stated predictive formulas alongside with the corresponding standard errors and *p*-values are given in Table 1.

## 3. Discussion

Currently one of the major aims of breast cancer research is to identify the set of EBC patients that are at high risk of cancer relapse, treat them more aggressively to eradicate residual tumor cells and follow up on these patients more closely. On the contrary it would be of considerable interest to identify other patients with negligible chance of relapse and minimize pre- or postsurgical systemic therapy to prevent occurrence of long-term toxicities (cardiac, pulmonary, hepatic, and secondary malignancies). We previously identified four oncomiRs and one tumor-suppressive miR that enable monitoring of EBC [10]. The notion of elevated levels of miR-155 [11] and miR-24 [6] in sera during pathogenesis of aggressive BC was independently confirmed. Here we utilized a suitable “GEE multivariate data analysis model” that is able to calculate expectation of the miR level in serum for each EBC patient at a particular time point. Using the GEE applied to our 133-patient cohort we discovered that two of the studied oncomiRs, miR-155 and miR-24, are highly predictive of BC relapse (Figure 1). To our knowledge, this is the first report that would indicate the relationship of miR-155 and miR-24 levels with the pathogenesis of the EBC relapse. Interestingly, while levels of miR-155 decreased gradually with minimum at the time point III the miR-24 declined already at time point II. The biology behind these phenomena is not fully understood although these differences may reflect differential stability or turnover of these two microRNAs. In addition, the dynamics of microRNAs could be partly influenced by the therapy-combination or germ line predisposition. Our data indicate that these variables including different therapies (chemotherapy, radiation and hormonal) or family history have not influenced the levels of miR-24 and miR-155 (*p*-values 0.55, 0.63, 0.60, 0.91 or 0.35, 0.17, 0.18, 0.80; respectively). Experimental studies previously suggested that miR-155 adds to the BC pathogenesis by promoting neo-angiogenesis via targeting the VHL gene and that the miR-155/VHL levels associate with the decreased overall survival of triple negative BC [12]. Another study associated miR-155 levels with decreased efficiency of homologous recombination repair and enhanced sensitivity to irradiation via targeting RAD51 expression [13]. Such possibility would indicate accelerated clonal evolution of cancer stem cells following the radiotherapy in miR-155-overexpressing BC patients. Thus, testing the EBC patients for miR-155 or miR-24 may be a useful tool to identify who will benefit from an irradiation-based therapy. In addition, miR-155 levels were shown highly indicative of the clinical response to aromatase inhibitors as one of its targets, Hexokinase 2 (HK2), is involved in the adaptation of tumor cells to aromatase inhibitors [14]. Furthermore, high miR-155-expressing tumors showed poorer prognosis upon hormonal treatment [14]. Much less is currently known on the molecular mechanisms of relapse mediated by elevated miR-24. One study indicated that miR-24 level correlates with BC lung metastasis via targeting the Prosaposin gene [15]. There are however indications that other studied oncomiRs (miR-19a [16], miR-181b [17]) may also be involved in molecular mechanisms preceding the relapse. Interestingly, all four studied oncomiRs (miR-155, miR-19a, miR-181b, and miR-24) modulate the TGFβ pathway albeit by targeting different mechanisms [18].

Of considerable interest is to combine the newly identified microRNA-based values with the cellular parameters identified within a tumor. Such combination may further stratify the clinical outcome of the EBC patients. Indeed, we found one such parameter, Ki-67 expression, which further specified the relapse probability defined by the levels of miR-155 or miR-24 in serum. Our data are supported by a study that revealed a positive correlation between Ki-67 positivity and miR-155 levels in the tumor cells [14]. Another explanation why Ki-67 associates with increased relapse rate is that it marks tumor cells with high proliferative capacity. Therefore, while previous study allowed us to identify 4 oncomiRs to monitor tumor burden in EBC patients, this updated work allowed us to further specify which of these microRNAs are also involved in the pathogenesis of the relapse. Additionally, while our previous work associated EBC risk factors with the microRNA profiles, this study suggests that only one of these risk factors, Ki-67, is associated with the relapse and makes the “prognostic index” even stronger (Figure 2). Although the newly presented data from 133 EBC patients will require subsequent additional validation studies in larger population, we strongly believe that GEE multivariate data analysis model of miR-155 and miR-24 determined in the patient sera (at time point II, together with Ki-67 positivity) allow relapse prediction and may, in the future, represent valuable approach for the EBC therapy stratification.

## 4. Materials and Methods

### 4.1. Patients

Patients with EBC (this includes invasive BC stages I, IIA, IIB and IIIA) donated the sera at the time points I–IV. The EBC relapse occurrence in the Czech population is relatively variable ranging between 5 and 35%. We have observed 13 cases of the relapsed EBC in our cohort, which represents 9.8%. The relapsed EBC was detected with combination of laboratory (CEA, CA 15-3), clinical (physical examination), and imaging (abdominal ultrasound, chest X-ray, CT scans, PET scan) approaches. The metastatic process was biopsy-verified from the suspected lesions. Median follow-up was 53.25 months (ranges 21.5–68.5). The Table 2 provides essential clinical information for each of the 133 patients including the age, menopausal status, progression-free survival, deaths, personal cancer history, histological types, tumor and node characteristics, tumor grade, hormonal receptor status, Ki-67, HER2 expression and risk groups. Positive family history on breast cancer was noted in 20 out of 133 patients (15%). Tumors were removed by partial surgery (segmentectomy, lumpectomy) or total mastectomy according to the size and localization of tumors. Regional lymphatics were probed by sentinel lymph node (SLN) biopsy; in case of SLN positivity the axillary lymphatics were removed. For all fit high-risk patients the adjuvant chemotherapy based on anthracyclines (4 cycles of doxorubicine and cyclophosphamide) and sequentially taxanes (4 cycles of docetaxel once per 3 weeks or 12 cycles of paclitaxel weekly) was applied. Trastuzumab treatment for up to 52 weeks was applied in HER2-positive patients. Radiotherapy followed national and international standard; briefly 50 to 66 Gy (2 Gy/fraction/5 days in a week) was recommended. Either chemotherapy or radiotherapy was applied to each patient. Hormonal therapy was applied according to the positivity of a specific receptor. In total, 73% (*N* = 97) patients were treated with radiotherapy, 31% (*N* = 42) with chemotherapy, and 88% (*N* = 118) with hormonal therapy. 21 healthy female-volunteers served as controls. Blood sera were collected and stored as reported previously [10]. RNA was isolated using miRNeasy® Mini Kit (Qiagen, Hilden, Germany) from 200 µL of sera (lysed by 1ml of QIAzol® Lysis reagent (Qiagen)) with previously reported modifications [10]. Reverse transcription and quantitative polymerase chain reaction provided the *C*_t_ values of the oncomiRs and let-7a in EBC vs. healthy sera (collected from 21 healthy female-volunteers, age ranges 25–60 years) using 2^−(Δ*C*t)^ equation in duplicate samples [10].

### 4.2. Ethics Approval and Consent to Participate

Patient sera were collected in years 2010–2014 from EBC (*N* = 133, median age 61.5) following the written informed consent based on the Helsinki declaration, and approved by the Ethics Committee of the Regional Hospital in Liberec, Czech Republic (under #EK/83/2010, 23.6.2010).

### 4.3. Statistical Analysis

Serum levels of the oncogenic miRs (miR-155, miR-19a, miR-181b, and miR-24, all relative to let-7a) are recorded for each BC patient at four time points (I–IV) during the therapy. Longitudinal multivariate data analysis was performed to stochastically model the serum levels of each of the miRs. In particular, we present the use of a possible extension of generalized linear models (GLM), namely the GEE method. All the classical regression approaches are based on the assumption that the serum levels for one patient in different time points are independent. However, this assumption can be sometimes unrealistic or at least questionable. GEE were introduced by Liang et al. in 1986 as a method for estimating model effects if the independence assumption is violated [19]. The following GEE model is used for the miRs:

$$\begin{array}{cc} E[miR_{i,t}] & =\exp\{\beta_{R}(Patient_{i}\;relapsed)+\beta_{N}(Patient_{i}\;not\;relapsed)+\beta_{I}(Patient_{i}\;in\;I)+\beta_{II}(Patient_{i}\;in\;II)\\ & +\beta_{III}(Patient_{i}\;in\;III)+\beta_{IV}(Patient_{i}\;in\;IV)\}] \end{array}$$

Here, *E*[*miR_i_*_,*t*_] is the expectation of the miR’s serum level *miR_i_*_,*t*_ for each BC patient identification *i* at time point *t* = *I*, *II*, *III*, *IV*. The distribution of the miR’s serum level is a Gamma distribution and the autoregression correlation structure of order one is chosen for the dependence modeling within each miR measurement over the time points. The mathematical syntax of expression, e.g., (*Patient_i_ in II*), is that it equals one if and only if the *i*th BC patient is at time point II; zero otherwise. The regression coefficients *β*’s represent the quantitative impact of the subsequent true/false indicator in the bracket. e.g., *β_R_* quantifies the effect of a patient being relapsed on the corresponding miR’s serum level. For more detailed practical statistical introduction to the GEE elsewhere [20].

## 5. Conclusions

The levels of miR-155 and miR-24 relate to EBC recurrence, particularly at the post-surgery time point, as they likely indicate size of the residual tumor burden (analyzed using the multivariate GEE). The acquisition of Ki-67 status specifies the relapse probability defined by miR-155 or miR-24 level. Usage of these prediction formulas calculated through the GLM multivariate data analysis model may represent a highly valuable tool for relapse detection in EBC patients to improve their clinical monitoring.

## Figures and Tables

**Table 1:** Prediction of the relapse’s probability: The estimated effects of the risk characteristics alongside with the corresponding standard errors and *p*-values (A) when only miR-155 values are considered; (B) when only miR-24 are considered; or (C) when both miR-155 and miR-24 values are considered. Negative estimated effect of the risk characteristic corresponds to higher predicted probability of relapse.

	Risk Characteristic	Estimate	Standard Error	*p*-Value
(A)	Intercept	4.019	0.715	<0.001
	miR-155	−0.428	0.194	0.028
	Ki67 ≥ 20%	−1.788	0.717	0.013
	Triple negative	−0.093	1.937	0.961
	HER2 positive	−0.536	1.865	0.774
	Grade III	−0.960	0.897	0.284
	Node positive	−0.113	1.035	0.913
	Chemotherapy (yes)	−1.738	1.163	0.135
	Radiotherapy (yes)	1.600	1.188	0.178
	Hormonal therapy (yes)	0.936	1.674	0.576
	Family history (yes)	−0.407	1.197	0.734
(B)	Intercept	5.023	1.038	<0.001
	miR-24	−1.311	0.565	0.020
	Ki67 ≥ 20%	−1.633	0.719	0.023
	Triple negative	1.444	2.287	0.528
	HER2 positive	−0.030	2.047	0.988
	Grade III	−0.991	0.875	0.258
	Node positive	−0.593	1.007	0.556
	Chemotherapy (yes)	−2.364	1.239	0.056
	Radiotherapy (yes)	1.526	1.174	0.194
	Hormonal therapy (yes)	1.682	1.968	0.393
	Family history (yes)	−0.177	1.161	0.879
(C)	Intercept	5.687	1.179	<0.001
	miR-155	−0.396	0.190	0.038
	miR-24	−1.206	0.591	0.041
	Ki67 ≥ 20%	−1.605	0.743	0.031
	Triple negative	0.681	2.331	0.770
	HER2 positive	−0.282	2.028	0.889
	Grade III	−1.197	0.949	0.207
	Node positive	−0.369	1.117	0.741
	Chemotherapy (yes)	−3.005	1.549	0.052
	Radiotherapy (yes)	1.709	1.337	0.201
	Hormonal therapy (yes)	1.298	1.939	0.503
	Family history (yes)	−0.109	1.269	0.932

**Table 2:** Clinical data of the early breast cancer (EBC) patient cohort including the patients’ characteristics, survival, histopathology and staging.

Clinical Parameters	Values
Number of patients	133
Number of tumors	134
Age	Median 61.5 (37–84) years
Follow-up	Median 53.25 (21.5–68.5) months
PFS (progression free survival)	Median 51.5 (11–67.5) months
Deaths due to cancer (overall deaths)	2 (6)
premenopausal	23
postmenopausal	110
Histology ductal	97 (72%)
Histology lobular	10 (8%)
Histology mixed + others	27 (20%)
Tumors in personal history	15 (11%)
pT1a (tumor 0.1–0.5 cm)	3 (2%)
pT1b (tumor 0.5–1.0 cm)	21 (16%)
pT1c (tumor 1.0–2.0 cm)	70 (52%)
pT2 (tumor 2.0–5.0 cm)	40 (30%)
pT3 (tumor > 5 cm)	0
pT4 (invasion to chest, skin, inflam. BC)	0
N+ (positive lymphatic nodes)	26 (19%)
N− (negative lymphatic nodes)	108 (81%)
G I (histological grade 1)	27 (20%)
G II (histological grade 2)	72 (54%)
G III (histological grade 3)	27 (20%)
No grade	8 (6%)
HR+ (positive for hormonal receptors)	119 (89%)
HR− (negative for hormonal receptors)	15 (11%)
HER+ (HER2 positive)	4 (3%)
HER− (HER2 negative)	125 (93%)
No HER	5 (4%)
Ki-67 0–19% of positive cells	100 (75%)
Ki-67 > 20% of positive cells	29 (21%)
No Ki-67 (negative)	5 (4%)
Triple negative (HR/HER2 negativity)	12 (9%)
Low-risk group	65 (49%)
High-risk group	68 (51%)

## Supplementary Materials

Supplementary materials can be found at www.mdpi.com/1422-0067/18/10/2116/s1.

## References

[1] Mulrane L., Klinger R., McGee S.F., Gallagher W.M., O’Connor D.P. (2014). microRNAs: A new class of breast cancer biomarkers. Expert Rev. Mol. Diagn..

[2] Zoon C.K., Starker E.Q., Wilson A.M., Emmert-Buck M.R., Libutti S.K., Tangrea M.A. (2009). Current molecular diagnostics of breast cancer and the potential incorporation of microRNA. Expert Rev. Mol. Diagn..

[3] Iorio M.V., Ferracin M., Liu C.G., Veronese A., Spizzo R., Sabbioni S., Magri E., Pedriali M., Fabbri M., Campiglio M. (2005). MicroRNA gene expression deregulation in human breast cancer. Cancer Res..

[4] Kim K., Chadalapaka G., Lee S.O., Yamada D., Sastre-Garau X., Defossez P.A., Park Y.Y., Lee J.S., Safe S. (2012). Identification of oncogenic microRNA-17–92/ZBTB4/specificity protein axis in breast cancer. Oncogene.

[5] Li S., Yang C., Zhai L., Zhang W., Yu J., Gu F., Lang R., Fan Y., Gong M., Zhang X. (2012). Deep sequencing reveals small RNA characterization of invasive micropapillary carcinomas of the breast. Breast Cancer Res. Treat..

[6] Wu Q., Wang C., Lu Z., Guo L., Ge Q. (2012). Analysis of serum genome-wide microRNAs for breast cancer detection. Clin. Chim. Acta.

[7] Kim S.J., Shin J.Y., Lee K.D., Bae Y.K., Sung K.W., Nam S.J., Chun K.H. (2012). MicroRNA let-7a suppresses breast cancer cell migration and invasion through downregulation of C–C chemokine receptor type 7. Breast Cancer Res..

[8] Song J., Bai Z., Han W., Zhang J., Meng H., Bi J., Ma X., Han S., Zhang Z. (2012). Identification of suitable reference genes for qPCR analysis of serum microRNA in gastric cancer patients. Dig. Dis. Sci..

[9] Zhao H., Shen J., Medico L., Wang D., Ambrosone C.B., Liu S. (2010). A pilot study of circulating miRNAs as potential biomarkers of early stage breast cancer. PLoS ONE.

[10] Sochor M., Basova P., Pesta M., Dusilkova N., Bartos J., Burda P., Pospisil V., Stopka T. (2014). Oncogenic microRNAs: miR-155, miR-19a, miR-181b, and miR-24 enable monitoring of early breast cancer in serum. BMC Cancer.

[11] Eichelser C., Flesch-Janys D., Chang-Claude J., Pantel K., Schwarzenbach H. (2013). Deregulated serum concentrations of circulating cell-free microRNAs miR-17, miR-34a, miR-155, and miR-373 in human breast cancer development and progression. Clin. Chem..

[12] Kong W., He L., Richards E.J., Challa S., Xu C.X., Permuth-Wey J., Lancaster J.M., Coppola D., Sellers T.A., Djeu J.Y. (2014). Upregulation of miRNA-155 promotes tumour angiogenesis by targeting VHL and is associated with poor prognosis and triple-negative breast cancer. Oncogene.

[13] Gasparini P., Lovat F., Fassan M., Casadei L., Cascione L., Jacob N.K., Carasi S., Palmieri D., Costinean S., Shapiro C.L. (2014). Protective role of miR-155 in breast cancer through RAD51 targeting impairs homologous recombination after irradiation. Proc. Natl. Acad. Sci. USA.

[14] Bacci M., Giannoni E., Fearns A., Ribas R., Gao Q., Taddei M.L., Pintus G., Dowsett M., Isacke C.M., Martin L.A. (2016). miR-155 drives metabolic reprogramming of ER+ breast cancer cells following long-term estrogen deprivation and predicts clinical response to aromatase inhibitors. Cancer Res..

[15] Ell B., Qiu Q., Wei Y., Mercatali L., Ibrahim T., Amadori D., Kang Y. (2014). The microRNA-23b/27b/24 cluster promotes breast cancer lung metastasis by targeting metastasis-suppressive gene prosaposin. J. Biol. Chem..

[16] Farazi T.A., Horlings H.M., Ten Hoeve J.J., Mihailovic A., Halfwerk H., Morozov P., Brown M., Hafner M., Reyal F., van Kouwenhove M. (2011). MicroRNA sequence and expression analysis in breast tumors by deep sequencing. Cancer Res..

[17] Bisso A., Faleschini M., Zampa F., Capaci V., De Santa J., Santarpia L., Piazza S., Cappelletti V., Daidone M., Agami R. (2013). Oncogenic miR-181a/b affect the DNA damage response in aggressive breast cancer. Cell Cycle.

[18] Serpico D., Molino L., Di Cosimo S. (2014). microRNAs in breast cancer development and treatment. Cancer Treat. Rev..

[19] Liang K., Zeger S.L. (1986). Longitudinal data analysis using generalized linear models. Biometrika.

[20] Hudecova S., Pesta M. (2013). Modeling dependencies in claims reserving with GEE. Insur. Math. Econ..

