# Supplementary material for: Prediction Potential of Serum miR-155 and miR-24 for Relapsing Early Breast Cancer
Source: Int J Mol Sci. 2017 Oct 10;18(10):2116. doi: 10.3390/ijms18102116 (PMC5666798; doi:10.3390/ijms18102116)
Supplement: Supplementary file 1 [file ijms-18-02116-s001.pdf]

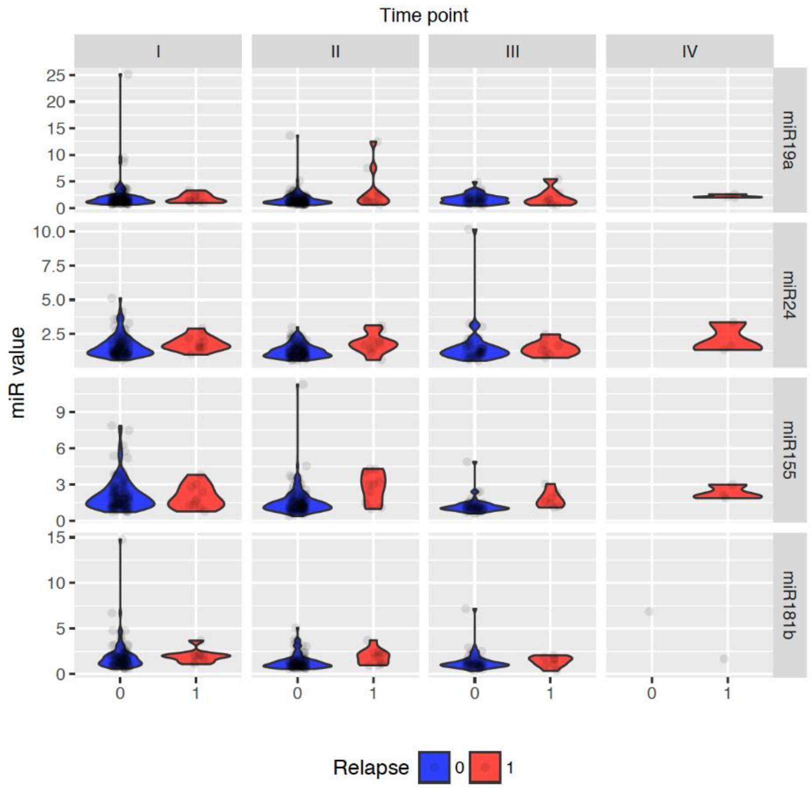

Figure S1. miR-155, miR-19a, miR-181b, and miR-24 levels at four time points I–IV, shown separately for the relapsed and non-relapsed patients.

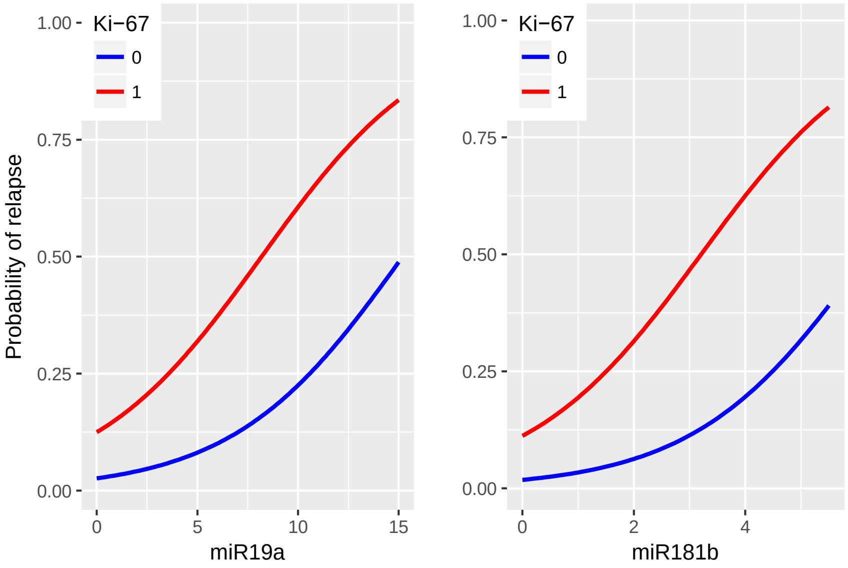

Figure S2. Predicted probability of relapse with respect to the values of miR-19a and miR-181b at time point II and according to Ki-67 positivity (>20% equals to Yes = 1, No = 0 equals to <20%) displayed together with 95% confidence bands. *p*-Value = 0.249 in case of miR-19a/Ki-67 and *p*-value = 0.240 in case of miR-181b/Ki-67.
